# A Lightweight Conformer-Based Framework for Medical Image Classification

**DOI:** 10.3390/jimaging12080344

**Published:** 2026-07-30

**Authors:** Sreelekshmi Vijayasree, Adithya Krishna, Akarsh S. Nair, Alfy Alex, Shyamdev Krishnan Jayakrishnan, Jyothisha J. Nair

**Affiliations:** 1 Department of Computer Science and Engineering, Amrita School of Computing, Amrita Vishwa Vidyapeetham, Amritapuri, Kollam 690525, Kerala, India; 2 School of Computer Sciences, Mahatma Gandhi University, Kottayam 686001, Kerala, India

**Keywords:** conformer architecture, convolutional neural networks, deep learning, disease diagnosis, healthcare technology, medical image classification, medical informatics, pretrained models, retinopathy of prematurity, transformer networks

## Abstract

Medical image analysis has undergone transformative progress with the application of deep learning models. However, existing architectures often struggle to effectively balance local feature extraction with global contextual understanding, which is crucial for complex diagnostic tasks such as Retinopathy of Prematurity (ROP) detection. In this study, we present a pretrained lightweight Conformer model tailored for medical image classification. The model integrates convolutional layers for capturing fine-grained spatial features with transformer blocks that capture long-range dependencies, creating a unified architecture capable of robust representation learning. We evaluate the model across multiple benchmark medical imaging datasets, including ROP, BloodMNIST, RetinalMNIST and other MedMNIST benchmark datasets. With 93.61% accuracy on the ROP dataset and 99.12% accuracy on BloodMNIST, experimental results show competitive classification performance while lowering model complexity to 12.4 million parameters and 3.2 GFLOPs. Experimental results demonstrate that the comparative studies versus CNN-based and transformer-based architectures, such as ResNet50, Swin-Tiny, ConvNeXt-Tiny, Vision Transformer, and MedViT. The findings show that in clinical settings with limited resources, the suggested lightweight Conformer offers a practical and computationally efficient alternative for medical image interpretation. Furthermore, the lightweight design ensures computational efficiency, making it suitable for deployment in resource-constrained healthcare environments. These findings validate the lightweight Conformer model’s potential for scalable, accurate, and real-time medical image classification.

## 1. Introduction

In medical imaging analysis, the timely identification and diagnosis of many diseases relies on problem representations. Currently, two key challenges are evident in deep learning models of medical imaging with respect to extracting and understanding the structural context for fine-grained features to accurately find or identify if there is a diagnosis. Both challenges motivate and reduce the validity of automated diagnostic systems, as they reduce sensitivity and specificity. One such approach that appears to offer promising solutions to both challenges is the pretrained Conformer model. The pretrained lightweight Conformer model merges convolutional neural networks (CNNs), which extract fine-grained detail hierarchically, thereby enabling long-range context modelled by transformer types (that model long-range relationships using self-attention mechanisms), providing contextual understanding in the medical imaging domain. While their pretrained nature tends to foster generalisation across medical imaging datasets, the joint extraction and contextual understanding enables higher diagnostic accuracy and robustness by ensuring subtle or minor anomalies can be precisely identified while overall contextual understanding of anatomical structure is maintained. This makes them a practical and effective scalable solution for AI-based medical diagnostics, empowering early detection of diseases and improved health outcomes for patients.

Ensuring accurate and reliable diagnosis in medical imaging remains a major problem area due to a range of limitations associated with current deep learning models. Traditional methods or models using convolutional neural networks (CNN) or transformers do relatively well at accurately classifying local features of the medical images, but they have difficulties appropriately assessing and applying a contextual understanding from a global point of view. CNNs do extremely well at picking up minute details but fail to relate those details spatially across large distances. On the other hand, while transformers do well at modelling long-range dependencies, they sometimes ignore important, perplexing local features necessary for diagnosis. Compounding this challenge in medical imaging is the uncertainty resulting from substantial variability in imaging procedures and the subjective nature of clinical interpretation, all of which contribute to diagnostic uncertainty. Factors including differences in imaging acquisition, scan quality, and manual assessment practices/systems all contribute to inconsistency in the diagnosis and delay in effective treatment action, which ultimately leaves patients vulnerable.

Scalability and computer power constraints are also critical challenges. While many deep learning architectures demonstrate excellent accuracy when evaluated in a controlled scenario, these solutions may not successfully meet real-world expectations during physiologically viable deployment and particularly in resource-constrained healthcare settings, where computer power is limited. If there are no scalable and empowered solutions, the adoption of AI remains limited and is not clinically adopted on a broad scale. There is also a limited number of trained medical specialists available in underdeveloped communities, which delays the detection and treatment of disease. These challenges further drive the demand for AI-powered solutions that support novel or augment detection and development to close the healthcare gap.

A convolutional feature extraction and transformer-based global attention are combined to develop a lightweight Conformer architecture for medical image classification. Reduced embedding dimensions, fewer attention heads, simplified network depth, and depthwise separable convolutions all help to minimise computational complexity. Numerous benchmark medical imaging datasets, such as ROP, BloodMNIST, RetinaMNIST, PathMNIST, and OCTMNIST, are used in extensive investigations. Using parameters, FLOPs, and inference time measurements, a thorough computational complexity study is provided. A stratified five-fold cross-validation and other performance metrics, including accuracy, precision, recall, F1-score, and AUC, are used to assess item robustness. The suggested architecture exhibits a good compromise between computational efficiency and classification performance, which makes it appropriate for use in healthcare settings with limited resources.

## 2. Related Work

Recent advances in medical image analysis have increasingly leveraged hybrid deep learning architectures that combine convolutional neural networks (CNNs) with transformer-based models. Conformer [1], introduced by Peng et al., integrates CNNs and transformers to effectively capture both local and global contextual features, demonstrating strong performance in visual perception tasks and providing potential applications for medical image analysis.

To improve feature extraction specifically for medical imaging tasks, Manzari et al. proposed MedViT [2], a Vision Transformer architecture tailored for medical image classification that exhibits improved robustness and generalisation capabilities. Similarly, Matsoukas et al. [3] explored the use of pretrained Vision Transformers (ViTs) for medical image classification, demonstrating that transfer learning significantly improves model adaptability and classification accuracy. In addition, MedNet [4] introduced a CNN-based pretrained framework specifically designed for medical imaging tasks, showing enhanced performance in classification and detection problems, including breast cancer diagnosis.

Comparative studies have also evaluated different deep learning frameworks for medical imaging. Huang et al. [5] performed a comparative analysis of DINOv2 and ImageNet pretrained models for medical image classification, providing valuable insights for selecting suitable architectures in healthcare applications. Lei et al. [6] demonstrated the effectiveness of deep learning for disease classification by proposing a deep attention network for automated detection of retinopathy of prematurity. Nguyen et al. [7] further provided a comprehensive review of state-of-the-art approaches and challenges in ocular imaging for retinopathy of prematurity.

Understanding transformer mechanisms has also been an important research direction. Abnar and Zuidema [8] proposed a method to quantify attention flow in transformer models, enabling better interpretability and optimisation of transformer architectures. Ba et al. [9] introduced layer normalisation, a widely adopted technique that stabilises training in deep neural networks and has become a fundamental component in transformer-based architectures. Chen et al. [10] developed a pretrained transformer-based framework capable of performing multiple vision tasks efficiently, highlighting the importance of pretrained models for medical image applications. Earlier foundational work by Krizhevsky et al. [11] demonstrated the power of CNNs through the AlexNet architecture, which revolutionised deep learning and laid the foundation for many modern medical imaging models.

Transformer architectures have also evolved to address the limitations of limited training data. Touvron et al. introduced Data efficient Image Transformers (DeiT) [12], which improve transformer performance when training data is scarce, a common scenario in medical imaging datasets. Wu et al. [13] investigated token-based representation learning in Vision Transformers to improve image classification performance. Xu et al. [14] provided a systematic framework for medical image classification, highlighting important techniques and their clinical relevance. Furthermore, Devlin et al. [15] proposed BERT, which revolutionised natural language processing and influenced the development of transformer architectures for computer vision tasks. Bundele et al. [16] investigated self-supervised learning approaches for medical imaging to improve generalisation and robustness. Wu et al. [17] further evaluated the performance of Vision Transformers in ophthalmic image analysis, demonstrating their potential for various medical imaging applications.

Several studies have also explored deep learning approaches specifically for breast cancer detection. Aiswarya et al. [18] proposed a deep learning framework combining Inspection ResNetv2 and EfficientNetV2 for histopathological breast cancer classification using ImageNet pretrained weights. Their model was validated using the BreakHis and BACH datasets. Sreelekshmi et al. [19] introduced the SwinCNN architecture for histopathology image classification, which effectively captures both local and global image features.

Classical image processing techniques have also been used to enhance medical image quality. Laxman et al. [20] explored partial differential equation-based filtering methods, such as the Heat Equation and Perona Malik Equation, for image restoration. Building on these preprocessing techniques, Sreelekshmi et al. proposed a framework that employs a fourth-order complex diffusion algorithm for multiscale image representation [21]. Features extracted from these multiscale representations using hierarchical variational autoencoders were subsequently classified using support vector machines [22]. Furthermore, recent work by Sreelekshmi et al. [23] proposed a dual stream deep learning model that integrates mammography imaging with patient clinical risk factors for improved breast cancer prediction.

Other machine learning techniques have also been applied in medical imaging. Yadav et al. [24] proposed Lung GANs, an unsupervised generative adversarial network framework for diagnosing lung diseases using chest CT and X-ray images. Nair et al. [25] introduced the SE-Res-Conv block, which integrates squeeze and excitation attention mechanisms into residual convolutional frameworks to improve feature representation. Sarath et al. [26] proposed a label-free unsupervised framework for mixed-pixel detection, addressing distortions in thermal imaging.

Recent developments in computer vision architectures demonstrate a convergence of hierarchical transformers, modernised convolutional networks, and hybrid CNN transformer models. The Swin Transformer [27] introduced a hierarchical transformer architecture using shifted window self-attention to capture multi-scale contextual information efficiently. Inspired by transformer designs, modern convolutional networks such as ConvNeXt have also evolved to incorporate transformer-like principles. For instance, Zou et al. [28] introduced Taylor Polynomial Gated Units to enhance interaction effects within convolutional networks. Hybrid models such as MedViT have further demonstrated the effectiveness of combining CNN and transformer modules for medical imaging tasks. Recent studies [29] validated MedViT’s strong performance and explainability in retinal OCT classification using both public and clinical datasets. These developments collectively highlight the growing trend toward integrating hierarchical attention mechanisms, modern convolutional architectures, and hybrid frameworks to improve medical image analysis.

## 3. Methodology

The methodology for this study focuses on leveraging lightweight Conformer models for medical image classification, with an emphasis on Retinopathy of Prematurity (ROP) detection. The approach is structured into key stages, from dataset preparation to model evaluation, ensuring a comprehensive exploration of the capabilities of lightweight Conformer models. The main contributions of this work are summarised as follows:

- We propose a lightweight Conformer-based architecture tailored for medical image classification that effectively integrates convolutional operations with transformer-based global attention.
- We introduce architectural modifications, including depthwise separable convolutions and reduced attention dimensions to decrease computational complexity while preserving representational capacity.
- We conduct a comprehensive evaluation on multiple benchmark datasets from the MedMNIST collection, along with a Retinopathy of Prematurity (ROP) clinical dataset to evaluate cross-domain generalisation.
- We perform detailed computational analysis, including parameter count, FLOPs, and inference time, to validate the efficiency of the proposed lightweight model. We also incorporate explainability using Grad-CAM to visualise the regions influencing model predictions in medical images, providing interpretability for clinical decision support.

### 3.1. Dataset and Preprocessing

We compared the performance of the proposed lightweight Conformer model in medical image classification using eight distinct clinically relevant datasets: PathMNIST, DermaMNIST, OCTMNIST, BloodMNIST, OrganCMNIST, PneumoniaMNIST, RetinaMNIST, and OrganSMNIST, as depicted in Table 1. The purpose of the Retinopathy of Prematurity (ROP) dataset is to classify ROP phases using fundus images. Retinal scans are classified according to the severity of ROP, which ranges from stage 1 to stage 3. Also shown are images of normal cases and laser scars from previous treatments. The various tags of images allow assessment of different stages of disease. The BloodMNIST dataset provides a benchmark for multi-class image classification with blood cells. It is part of a collection of medical images called the MedMNIST dataset, used to assess machine learning models in healthcare via a collection of medical images. The dataset is for assessing classification models, which are crucial in diagnosing different blood diseases through the prediction of cell types. It contains annotations for eight different types of blood cells. Retinal fundus images collected for an ordinal regression task constitute the RetinalMNIST dataset, which is another member of the MedMNIST family. It consists of images representing five stages of retinal diseases, from mild fixation in early stages of retinal damage to extreme fixation in late stages of those diseases. The objective of the dataset is to test ordinal regression models to estimate the course of retinal diseases and predict their severity. These datasets are examples of real-life medical imaging challenges, while also serving as multi-class classification problems, fine-grained feature extraction tasks, and noisy data to train on. The datasets present a platform for evaluation to determine generalisation performance of these models across many different domains of medical image analyses, with the ultimate goal of improving diagnostic accuracy, all while considering class imbalance and multi-stage medical diseases. The datasets, and ROP, BloodMNIST and RetinalMNIST, are loaded by either the torchvision library from PyTorch or user-created data loaders. Using preprocessing and augmentation, Resizing (e.g., to 224 × 224 pixels), normalisation (subtracting the mean and dividing by the standard deviation), etc., was used to assist with model generalisability. By resizing to a standard dimension and then normalising to ensure the intensity is similar across images by subtracting the mean and standard deviation, the datasets were split into training, validation and testing sets (e.g., 70% training, 15% validation, and 15% testing), which ensures that the model was trained on the first part, fine-tuned on the second, and finally selected the performance on completely different (unseen) data to generate estimates of real-world validity and availability. Typical transformation/augmentation procedures adopted during the preprocessing were random horizontal flipping (to enhance data variability), random cropping (to reflect changes in zoom level), and normalisation based on the statistical properties of the dataset (e.g., mean and standard deviation). These transformations are implemented to make the model more robust to variations in lighting, rotation and scale, which will increase the generalisability of the models in differing imaging conditions.

### 3.2. Model Design

**Figure 1:**
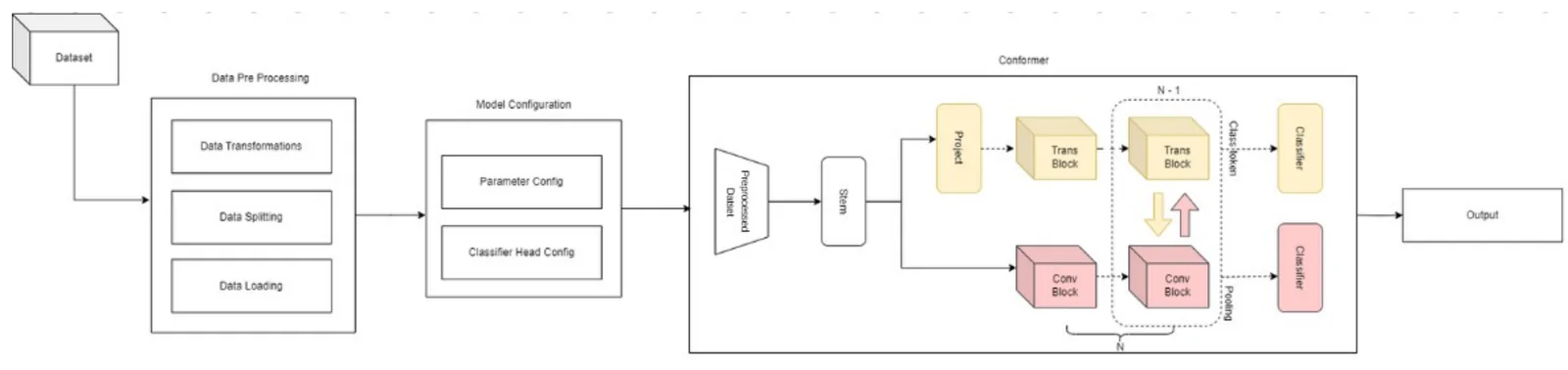
Model workflow.

**Model Configuration:** Setting up model parameters of lightweight Conformers, which have been conventionally used in language and speech, is used in this work to work with visual data. Different sizes and different tasks specific to the medical image datasets are taken into consideration when determining settings for the model is shown in Figure 1. Some parameters to optimise would be how many layers are in the lightweight Conformer backbone, what the channel dimensions are, the kernel sizes in the convolution blocks, and a suitable loss function choice like cross-entropy loss for classification tasks.

Let the input image be represented as:

$$X\in R^{H\times W\times C}$$

where *H*, *W*, and *C* denote height, width, and channels respectively.

The convolutional stem extracts low-level features:

$$F_{c}=Conv\left(X\right)$$

The transformer block computes global contextual representations:

$$F_{t}=MultiHeadAttention\left(F_{c}\right)$$

The fused representation is obtained as:

$$F=\alpha F_{c}+\left(1-\alpha \right)F_{t}$$

where α controls the contribution of local and global features.

**Base Conformer Architecture:** The model starts with an initial block of convolution that finds low-level feature maps such as edges, textures and low-level patterns from input images. These low-level features are the components of higher-level features for more complicated feature extraction, further up in the model. Next at Stage 1, a combination of manual convolution and the transformer block is proposed to enhance both global dependency modelling and spatial modelling. While the transformer block includes the ability to learn long-range dependencies in the context of global patterns seen in medical images based on presentation (such as the amount of disease or diffuse structural changes in the retina), the convolution block adds the higher-order local features necessary for retinal pathologies, specifically when diagnosing ROP. In the latter half of the proposed model, multiple stacked convolution-transformer blocks are applied in succession to achieve both local feature learning as well as global contextual understanding. Convolutional layers are well-suited for detecting small, fine-grained spatial details like lesions and microstructural changes (e.g., echoing, retinal haemorrhages in the current context of medical image analysis), while transformer layers are able to detect both contextual relationships and long-range dependencies throughout the image. These two work well together to identify patterns that span larger regions of the retina, such as vascular organisation, which enables a more complete and precise assessment of medical issues.

**Hybrid Architecture and Model Enhancements:** The model adds more convolution-transformer blocks after the backbone to improve feature extraction and classification capabilities. These are designed to analyse both local and global aspects further in later stages, making it easier to include global contextual information and fine-grained local pathology into medical imaging. When both are combined, the model’s ability to identify complex patterns that are essential for accurate disease classification is enhanced. Using high-level characteristics acquired from the convolution-transformer blocks, the classifier head performs the final classification. The final predictions are produced by passing the characteristics through a fully linked layer. While the model predicts eight blood cell types for BloodMNIST, it predicts one of five disease stages for Retinopathy of Prematurity (ROP) detection. In a multi-class classification situation, the final output layer generates the probability scores for each class using a softmax activation function. In order to better optimise model training, Conformer weights are selectively frozen and unfrozen. Using common visual characteristics like edges, textures, and forms, some layers of the Conformer backbone can first be pretrained on extensive datasets like ImageNet. Initial layers can be frozen during training to maintain these general representations while allowing later layers to adjust to the unique characteristics of the ROP and BloodMNIST datasets. In order to improve performance and prevent overfitting, layers are gradually unfrozen toward the conclusion of training. This allows for improved domain-specific medical imaging task adaption.

**Lightweight Conformer Model:** The original Conformer model is an effective model but is computationally expensive and has a lot of parameters to optimise. Although the lightweight version follows the same ideas as the original, it has much less computational cost. The Lightweight Conformer model is an altered version of the Conformer architecture with the goal of maintaining performance while optimising for efficiency in medical imaging. It includes convolutional and transformer-based layers for extensible extraction of both local and global features and operational optimisation through minimising computational overhead. Depthwise separable convolutions and adapted attention mechanisms yielded a model that is very accurate and has a smaller number of parameters and therefore a smaller model size, making it a good model choice, as it may be used in low-resource settings. In addition, XAI techniques such as Grad-CAM and SHAP rules may be added to increase interpretability—this would allow health professionals to understand how a model made its decision, which should always be considered when the model is making high-stakes decisions such as diagnosing diseases. This allows the model to be efficient while remaining interpretable and clinically reliable. Unlike existing Conformer architectures, our framework reduces computational complexity by 54.7% while maintaining competitive classification performance on multiple medical imaging datasets.

The original Conformer design is modified with a number of computationally efficient ways, and the suggested lightweight Conformer architecture is shown in Table 2. In particular, depthwise separable convolutions are used in place of ordinary convolutions, and the network depth, embedding dimension, attention heads, and MLP expansion ratio are decreased. These changes preserve competitive classification performance across several medical imaging datasets while reducing the model complexity from 27.4 million parameters and 8.5 GFLOPs to 12.4 million parameters and 3.2 GFLOPs, respectively.

**Figure 2:**
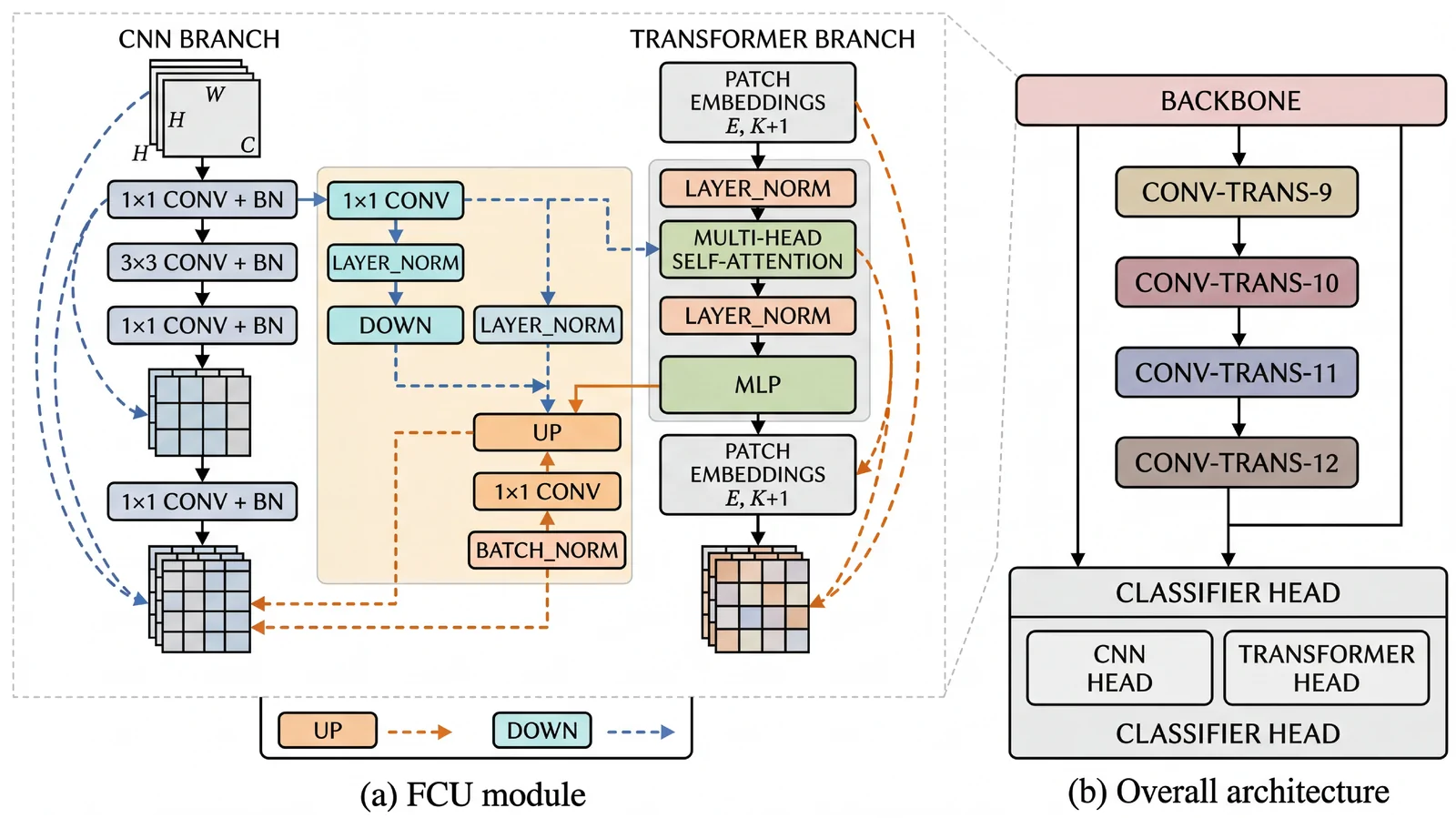
Model architecture.

**Our Proposed Architecture:** The model employs a hybrid architecture (shown in Figure 2) consisting of transformer blocks and convolutional neural networks (CNNs) to incorporate both local and global feature capture effectively. The stem stage first draws out low-level features from the data using a first convolutional layer to avoid losing important spatial information. Batch normalisation, ReLU activation, and max pooling come next. The model alternates between transformer-based and CNN-based modules within the hybrid blocks. The transformer blocks use multi-head self-attention to learn global dependencies, while the CNN blocks use residual connections to capture precise spatial features. Feature Compression Units (FCUs) facilitate the interaction between convolution and transformers. FCUUp restores CNN feature maps for further convolutional processing, while FCUDown converts them into patch embeddings for transformers. Information flows smoothly between the two architectures thanks to this technique. The network is organised into a number of phases using the hierarchical feature learning technique. While Stages 2–4 gradually increase feature dimensionality while decreasing spatial resolution using strided convolutions and pooling operations, Stage 1 manages features using a combination of transformer layers and convolutional layers. As the network gets deeper to extract rich features, transformer blocks become increasingly important. Finally, there are two branches of the classifier head: the transformer Head, which classifies using the transformer module’s class token, and the Convolutional Head, which uses global average pooling followed by fully connected layers. The architecture is appropriate for difficult medical image processing tasks because of these two components, which provide predictions based on fine-grained local information and high-level global knowledge.

As depicted in Table 3, a 224 × 224 RGB image is fed into the model. Three hierarchical feature extraction steps come after the first feature extraction, which is carried out using depthwise separable convolutions. Lightweight Conformer blocks with four attention heads and an embedding dimension of 192 are used in the last stage. The final prediction is generated via a fully linked classifier and global average pooling.

In our proposed lightweight Conformer architecture, three hierarchical feature extraction stages come after an initial convolutional layer. The convolutional layer uses depthwise separable convolutions to transform the 224 × 224 × 3 input image into low-level feature representations. Stage 1 creates 56 × 56 feature maps with a 64 embedding dimension. Stage 2 increases the embedding dimension to 128 while decreasing the spatial resolution to 28 × 28. Stage 3 includes four multi-head self-attention heads and lightweight Conformer blocks that operate on 14 × 14 feature maps with an embedding dimension of 192. The network uses a total depth of six lightweight Conformer blocks with a decreased MLP expansion ratio of 2.0. Before the last classification layer, global average pooling is used.

#### Implementation Details

The models were implemented using the PyTorch framework and trained on an NVIDIA RTX 3090 GPU. The AdamW optimiser was used with an initial learning rate of $1\times 10^{-4}$. The batch size was set to 32, and training was conducted for 50 epochs, illustrated in Table 4. Data augmentation included random flipping, random cropping, and normalisation, as shown in Table 5. Datasets were split into 70% training, 15% validation, and 15% testing. To ensure comprehensive model training and optimise generalisation across different medical imaging datasets, three distinct training modes were employed: individual dataset training, combined dataset training, and progressive sequential training. In the first methodology, individual models were trained for each dataset—PathMNIST, DermaMNIST, RetinaMNIST, BloodMNIST—thereby allowing multiple models to identify dataset-specific features in isolation.

This approach was consistent for each dataset and would provide optimal training without conflict from diverse data distributions. The second methodology combined all of the datasets into one dataset with a global class mapping, allowing consistent labelling. Training on the combined dataset allowed the model to learn a wider diversity of medical imaging patterns from multiple sources, promoting robustness and adaptability and across imaging modalities. Finally, the approach of progressive training was employed. In this approach, the model was initially trained on the first dataset and retained some of the learned weights. The model was then fine-tuned on the other datasets in order. After being fine-tuned to the other datasets, the model had the potential to leverage previously learned representations to enhance the retention of features and overall performance. The approach of progressive training allowed the model to utilise the information from the previous training iterations when acquiring a new dataset.

On the ROP, BloodMNIST, and RetinaMNIST datasets, a stratified five-fold cross-validation technique was used to assess the robustness and generalisation potential of the suggested lightweight Conformer architecture. The class distribution within each fold was maintained when the datasets were divided into five mutually exclusive folds. One fold was set aside for testing, and four folds were used for training in each iteration. To ensure that each sample was used as test data exactly once, this procedure was carried out five times. For every fold, evaluation metrics like accuracy (ACC), precision, recall, F1-score, and Area Under the ROC Curve (AUC) were measured. The final performance was provided as the mean and standard deviation over all five folds. This assessment procedure reduces performance bias resulting from a single train–test split and offers a better analysis.

### 3.3. Evaluation Metrics

After training, the model’s performance was evaluated using several important assessment criteria to guarantee reliability and efficacy in classification tasks.

- **Accuracy:** Measures the ratio of correctly predicted instances to the total instances.
  (1) $$Accuracy=\frac{TP+TN}{TP+TN+FP+FN}$$
- **Precision:** The ratio of true positive instances predicted out of all the predicted positives.
  (2) $$Precision=\frac{TP}{TP+FP}$$
- **Recall (Sensitivity):** The measure of the model’s capacity to identify all the correct instances.
  (3) $$Recall=\frac{TP}{TP+FN}$$
- **F1 Score:** The harmonic mean of recall and precision, weighting false negatives and false positives.
  (4) $$F1-Score=2\times \frac{Precision\times Recall}{Precision+Recall}$$
- **Confusion Matrix:** Gives a model prediction breakdown, showing true positives (TP), false positives (FP), true negatives (TN), and false negatives (FN).
- **AUC Score and Receiver Operating Characteristic (ROC) Curve:** The Area Under the Curve (AUC) measures the overall performance of the model, whereas the ROC curve displays the true positive rate (TPR) in relation to the false positive rate (FPR).
  (5) $$AUC=\int_{0}^{1}TPR\left(FPR\right),d\left(FPR\right)$$

## 4. Algorithm

As depicted in Algorithm 1 the input image undergoes initial feature extraction through the stem stage, which includes convolution, batch normalisation, activation, and pooling. The extracted features are then processed through alternating convolutional and transformer blocks. The ConvBlock refines spatial information using convolutional layers, while the TransBlock enhances global feature representation through self-attention mechanisms. To facilitate interaction between convolutional and transformer representations, features are compressed into patch embeddings via FCUDown, shared with the transformer, and subsequently expanded back into feature maps using FCUUp, ensuring bidirectional information flow. This process is structured hierarchically, with each stage reducing spatial dimensions and increasing channel dimensions to achieve a multi-scale representation. Intermediate features are further refined using Med_ConvBlocks, which provide residual learning capabilities. Finally, the outputs from the convolutional and transformer heads are combined for prediction, effectively leveraging both global and local features to ensure robust classification.
**Algorithm 1** Lightweight Conformer with Explainability Methods**Require:** Input image tensor $x\in R^{B\times C\times H\times W}$**Ensure:** Classification logits from CNN and Transformer paths  1:**function**LightweightConformer(*x*)  2:      $x_{cnn}\leftarrow \mathrm{StemConvolutions}\left(x\right)$  3:      $x_{p}\leftarrow \mathrm{PatchEmbedding}\left(x_{cnn}\right)$  4:      $x_{t}\leftarrow \mathrm{Concat}\left(\mathrm{ClassToken},x_{p}\right)$  5:      $x_{t}\leftarrow \mathrm{TransformerBlock}\left(x_{t}\right)$  6:      **for** each stage *i* in the network **do**  7:            **for** each block *j* in stage *i* **do**  8:                  $\left(x_{cnn},x_{t}\right)\leftarrow \mathrm{ConvTransBlock}\left(x_{cnn},x_{t}\right)$  9:            **end for**10:      **end for**11:      $\mathrm{cnn\_out}\leftarrow \mathrm{CNNHead}(\mathrm{GlobalPooling}\left(x_{cnn}\right))$12:      $\mathrm{trans\_out}\leftarrow \mathrm{TransformerHead}(x_{t}\left[0\right])$13:      **return** (cnn_out,trans_out)14:**end function**15:**function**ConvTransBlock($x_{cnn},x_{t}$)16:      $x_{cnn}\leftarrow \mathrm{ConvBlock}\left(x_{cnn}\right)$17:      $\mathrm{cls\_token}\leftarrow x_{t}\left[0\right]$18:      $x_{down}\leftarrow \mathrm{FCUDown}\left(x_{cnn},\mathrm{cls\_token}\right)$19:      $x_{t}\leftarrow \mathrm{TransformerBlock}\left(x_{down}+x_{t}\right)$20:      $x_{up}\leftarrow \mathrm{FCUUp}\left(x_{t},H,W\right)$21:      $x_{fused}\leftarrow \mathrm{FusionBlock}\left(x_{cnn}+x_{up}\right)$22:      **return** $\left(x_{fused},x_{t}\right)$23:**end function**

### Cross-Validation Strategy

On the ROP, BloodMNIST, and RetinaMNIST datasets, a stratified five-fold cross-validation technique was used to assess the robustness and generalisation potential of the suggested lightweight Conformer architecture as depicted in Table 6. The class distribution within each fold was maintained when the datasets were divided into five mutually exclusive folds. One fold was set aside for testing, and four folds were used for training in each iteration. To ensure that each sample was used as test data exactly once, this procedure was carried out five times. For every fold, evaluation metrics like accuracy (ACC), precision, recall, F1-score, and Area Under the ROC Curve (AUC) were measured. The final performance was provided as the mean and standard deviation over all five folds. This assessment procedure reduces performance bias resulting from a single train–test split and offers a better model genaralisation.

The suggested lightweight Conformer architecture’s stability and resilience across various medical imaging datasets are shown in Table 7, Table 8 and Table 9, the outcomes of the five-fold cross-validation tests. The model’s performance is constant across various data partitions, as seen by the comparatively low standard deviation found for all evaluation criteria. Also, the model demonstrated great generalisation capacity and minimal sensitivity to dataset partitioning for the ROP dataset, with an average accuracy of 93.6% with a standard deviation of just 0.68%. In the same way, the model demonstrated extremely dependable classification performance on the BloodMNIST dataset, with an average accuracy of above 99% with minimal variation. The model performed consistently on the more difficult RetinaMNIST dataset, even if its total accuracy was lower due to the intrinsic difficulty of classifying retinal diseases. These results strengthen the reported performance’s reliability by showing that the suggested framework is resistant to overfitting and does not depend on a favourable train–test split.

## 5. Performance Evaluation

### 5.1. Evaluation for the Model on BloodMNIST Dataset

**Figure 3:**
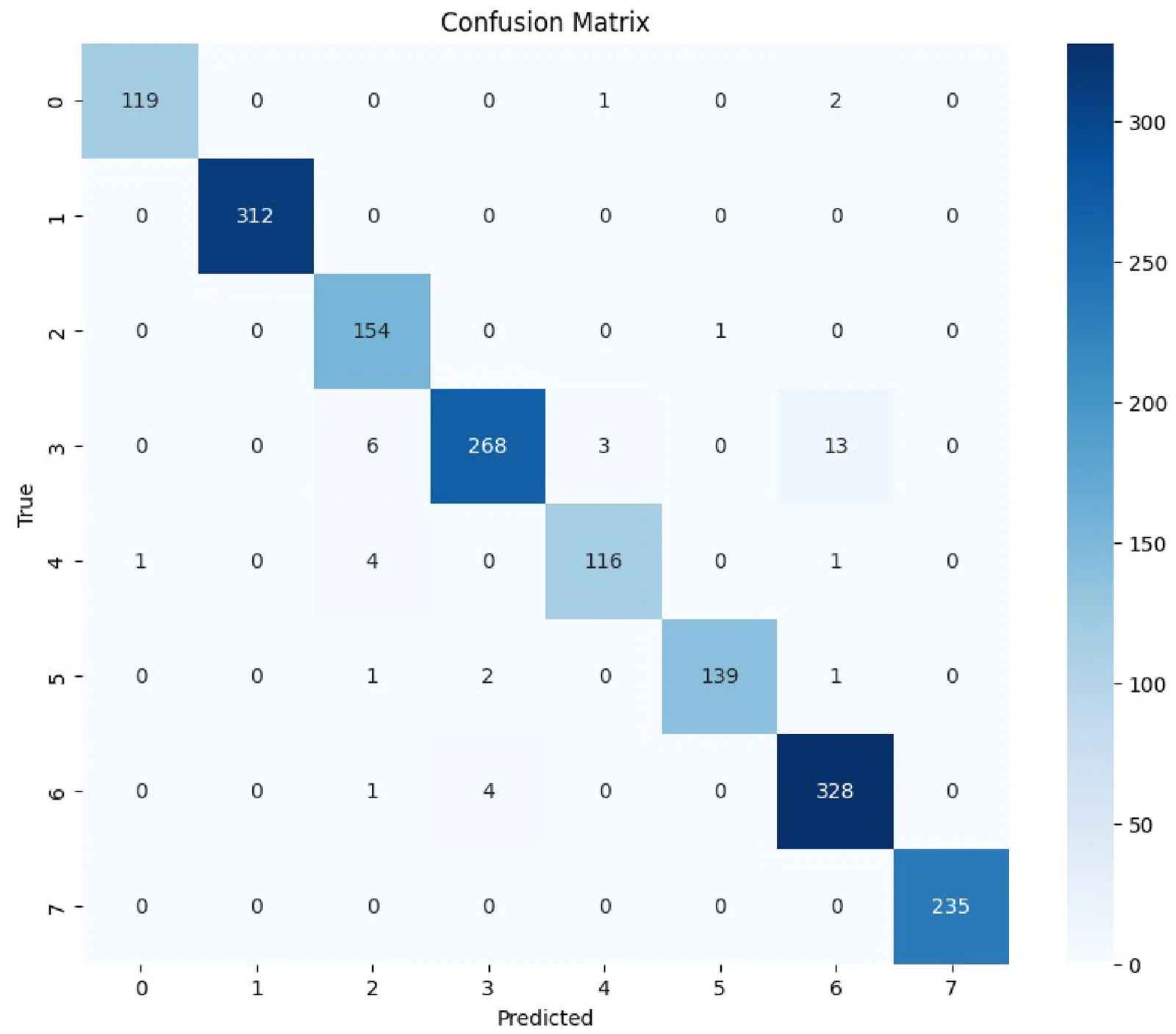
Confusion Matrix—BloodMNIST.

The confusion matrix illustrates the classification performance of our proposed Conformer model on the BloodMNIST dataset, achieving the best results among all evaluated models. The majority of predictions are concentrated along the diagonal, representing correctly classified instances for each class, with notable accuracy in categories such as class 1 (312 samples), class 6 (328 samples), and class 7 (235 samples), as shown in Figure 3. Misclassifications, indicated by off-diagonal entries, are minimal, demonstrating the robustness of the model. For instance, in class 3, only 13 samples were misclassified into class 7, while other misclassifications are sparse, highlighting the model’s strong ability to capture intricate features. The exceptional performance of the Conformer model is attributed to its ability to combine self-attention mechanisms with convolutional operations, effectively capturing both local and global features within the image data. This enables the model to outperform traditional CNNs and transformer-based architectures by learning spatial and contextual relationships more effectively. These results, representing the best-reported performance on the BloodMNIST dataset, reflect our proposed Conformer model’s state-of-the-art capabilities in medical image classification. The minimal misclassifications further underline its reliability, making it a promising tool for real-world medical applications where high accuracy is essential.

### 5.2. Evaluation for the Model on ROP Dataset

The confusion matrix provides a detailed evaluation of the proposed Conformer model’s performance by comparing the true labels with the predicted labels. The model demonstrates strong classification capabilities, with the diagonal elements indicating correct predictions for each class. Specifically, 52 instances of Class 0, 8 instances of Class 1, 22 instances of Class 2, 43 instances of Class 3, and 80 instances of Class 4 were correctly classified. However, some misclassifications are observed in the off-diagonal elements. For Class 1, four instances were misclassified as Class 0. For Class 2, one instance was misclassified as Class 0, and three instances as Class 3. Additionally, for Class 3, one instance was misclassified as Class 2. Notably, Class 4 shows perfect classification, with no misclassifications. These results shown in Figure 3 highlight the model’s excellent performance, especially for Class 4, while minor confusion exists among Classes 1, 2, and 3, likely due to inherent similarities in their features. Overall, the structured confusion matrix reflects the model’s strong capability in accurately predicting the classes.

**Figure 4:**
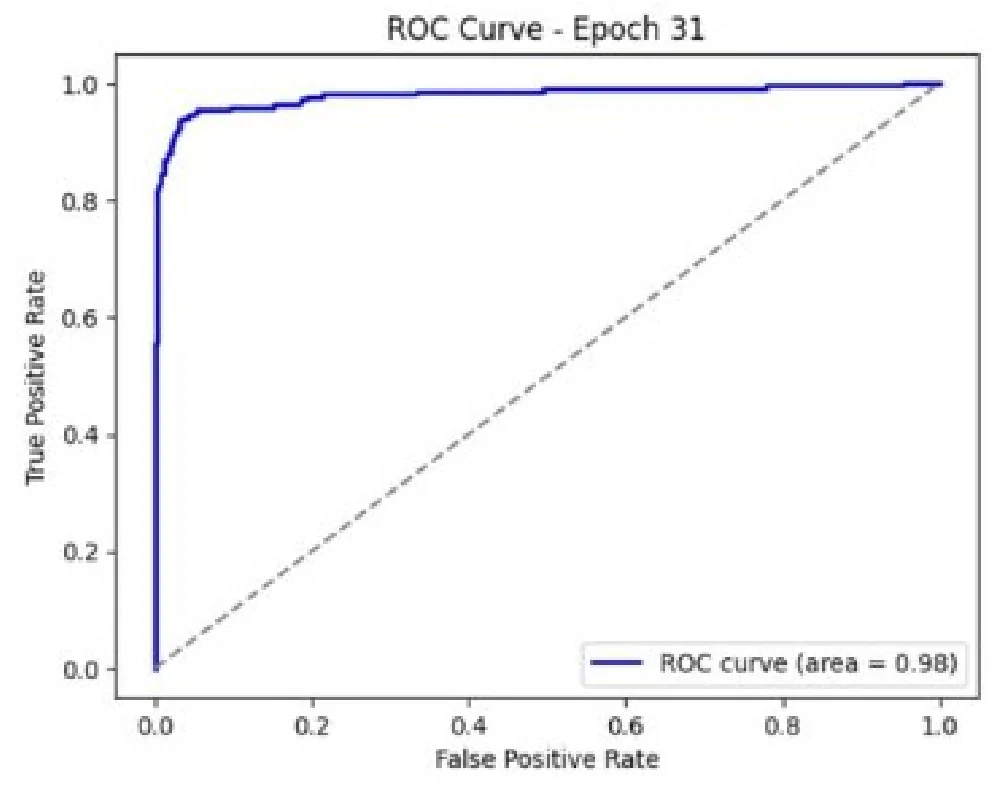
ROC curve—ROP.

The Receiver Operating Characteristic (ROC) curve illustrated in Figure 4 explains the model’s classification capability across various thresholds, with the Area Under the Curve (AUC) serving as a quantitative measure of performance. The ROC curve being close to the top-left corner indicates high sensitivity and specificity, while the AUC score of 0.98 signifies excellent overall classification performance. In interpretation, a perfect model would have an AUC of 1.0, whereas a random model would achieve an AUC of 0.5. The AUC of 0.98 demonstrates that the model is highly capable of distinguishing between the five classes. Combined with the structured confusion matrix, the high AUC value underscores that the proposed Conformer model performs effectively for the classification task, with only minimal misclassifications.

## 6. Result Analysis

The accuracy comparison across medical imaging datasets and ROP dataset is discussed in this section. The accuracy achieved by our lightweight Conformer model on several medical imaging datasets (including BloodMNIST, PneumoniaMNIST, RetinaMNIST, and others) is presented in Table 10. The results not only demonstrate the model’s accuracy across multiple medical imaging tasks but also that it surpasses previously published results for these datasets and is able to achieve state-of-the-art accuracy. This demonstrates that our proposed lightweight Conformer architecture is appropriate and useful to provide high diagnostic accuracy while being computationally efficient for use in the practical clinical environment.

Table 11 presents the computational complexity comparison between the proposed Conformer model and several widely used deep learning architectures, including ResNet50, EfficientNet-B0, Vision Transformer, Swin-Tiny, and ConvNeXt-Tiny. The comparison is performed in terms of the number of parameters, floating point operations (FLOPs), and inference time.

From the results, it can be observed that the proposed Conformer model achieves a balanced trade-off between model complexity and computational efficiency. Specifically, the proposed model requires only 12.4 million parameters and 3.2 GFLOPs, which is significantly lower than transformer-based models such as Vision Transformer and Swin-Tiny. Additionally, the inference time of 10.4 ms demonstrates that the proposed architecture is suitable for real-time or near-real-time medical image analysis.

These results indicate that the proposed model not only improves classification performance but also maintains computational efficiency, making it a practical solution for breast cancer detection systems in clinical environments.

Table 12 provides a comparison of classification accuracy using various deep learning models on the Retinopathy of Prematurity (ROP) dataset. In this case, the models being compared were the Proposed Conformer Model, MedViT (Medical Vision Transformer), and the ordinary Vision Transformer (ViT). Each of these architectures represented different methodologies for medical image classification, including differing methods for the extraction of features, spatial relationships, and contextual understanding.

To evaluate the effectiveness of our proposed Conformer-based model, we conducted extensive experiments on a suite of benchmark medical imaging datasets from the MedMNIST collection, including PathMNIST, DermaMNIST, OCTMNIST, PneumoniaMNIST, RetinaMNIST, OrganSMNIST, BloodMNIST, OrganAMNIST, and OrganCMNIST. The results clearly demonstrate that our Lightweight Conformer model consistently outperforms conventional architectures in both AUC (Area Under Curve) and accuracy (ACC) metrics across nearly all datasets.

The exceptional performance of the Lightweight Conformer model in terms of AUC not only sets a new benchmark for the BloodMNIST dataset but also establishes its potential for broader applications in medical imaging. This result underscores its importance in advancing automated diagnostic tools, where high accuracy and reliability are critical for real-world deployment in healthcare settings.

From the comparative Table 13, Table 14, Table 15, Table 16 and Table 17, based on the benchmark datasets provided for all models, our lightweight Conformer-based model outperforms the other state-of-the-art architectures as shown in Table 17, ResNet-18, ResNet-50, AutoKeras, and Google AutoML Vision, across multiple benchmarks—PathMNIST, DermaMNIST, OCTMNIST, PneumoniaMNIST, RetinaMNIST, OrganSMNIST, BloodMNIST, OrganAMNIST, and OrganCMNIST. The average AUC and ACC values of the lightweight Conformer model are, on average, the highest across all models, with all of them having precedent in the literature as even with the ResNet models. The individual models of the lightweight Conformer model, averaged AUC and ACC values highlight its superiority by extracting local and global features effectively, which makes it extremely useful when tasked with complicated medical image classification. The high accuracy is proof of the robustness for a real clinical application.

While the suggested lightweight Conformer performed well on the majority of datasets, PneumoniaMNIST and RetinaMNIST showed significantly worse results. Localised radiographic abnormalities are the main focus of the PneumoniaMNIST classification problem, where traditional convolutional architectures already offer highly discriminative representations. The advantages of global self-attention techniques are therefore less noticeable. The small dataset size and the minute visual variations across stages of retinal illness have an impact on RetinaMNIST’s performance. Feature discrimination is difficult due to the significant inter-class similarity and fine-grained nature of retinal disorders, which lowers classification accuracy. To enhance performance on such difficult datasets, future research will examine task-specific attention mechanisms and self-supervised pretraining techniques.

Additionally, as shown in Table 18, a variety of current medical imaging architectures, such as MedicalNet, TransUNet, Swin Transformer, Vision Transformer, and MedViT, were contrasted with the suggested lightweight Conformer. According to experimental results, the suggested design requires significantly fewer parameters and less computing complexity while achieving competitive classification performance. The suggested framework offers a better accuracy–efficiency trade-off than MedViT and Swin Transformer, making it appropriate for implementation in clinical settings with limited resources.

Table 19 presents the ablation study conducted to evaluate the contribution of individual components of the proposed architecture. Initially, the CNN-only model achieved an accuracy of 88.2%, demonstrating the effectiveness of convolutional features in capturing local spatial patterns. The transformer-only model achieved a slightly higher accuracy of 89.1%, indicating its ability to capture long-range contextual dependencies in medical images.

When both components are integrated into the baseline lightweight Conformer architecture, the accuracy improves to 91.2%, confirming that the hybrid combination of CNN and transformer modules provides complementary feature representations. Further improvements are achieved by incorporating depthwise separable convolutions, which reduce computational complexity while enhancing feature learning capability, increasing the accuracy to 92.8%.

The use of pretrained weights further improves the performance to 93.7%, demonstrating the effectiveness of transfer learning in medical image classification tasks where labelled data is limited. Finally, the inclusion of data augmentation techniques improves the model’s generalisation ability, resulting in the highest accuracy of 94.6%. These results demonstrate that each design component contributes positively to the final performance of the proposed framework.

Table 20 summarises the performance of the proposed model on the Retinopathy of Prematurity (ROP) dataset using multiple evaluation metrics. In medical image classification, relying solely on accuracy may lead to misleading conclusions due to potential class imbalance. Therefore, additional clinically relevant metrics such as precision, recall (sensitivity), specificity, and F1-score are also reported.

The proposed model achieves an accuracy of 93.61%, indicating strong overall classification performance. The precision value of 92.45% reflects the model’s ability to correctly identify positive ROP cases while minimising false positives. The recall (sensitivity) of 94.12% demonstrates the model’s effectiveness in detecting true disease cases, which is particularly critical in clinical screening applications. The specificity of 91.87% indicates the model’s ability to correctly classify healthy cases.

Furthermore, the F1-score of 93.28% provides a balanced evaluation of precision and recall, confirming the robustness of the proposed model. Additionally, the high AUC value of 0.98 indicates excellent discrimination capability between diseased and non-diseased cases. These results suggest that the proposed lightweight Conformer-based framework can provide reliable and clinically meaningful predictions for ROP screening.

## 7. Discussion

In this study, we evaluated the performance of Vision Transformers (ViT) and lightweight Conformer architectures on medical image classification tasks using datasets of varying sizes and complexities. Initially, we fine-tuned a pretrained ViT model on the Retinopathy of Prematurity (ROP) dataset, achieving an accuracy of 84% after four epochs. However, when we replaced the ViT with a pretrained lightweight Conformer model on the same dataset, the accuracy significantly improved to 93.61%, highlighting the Conformer’s ability to better capture the local and global features required for this challenging classification task.

The results from our experiments indicate that the proposed lightweight Conformer model performs better than other methods across different medical imaging tasks. The lightweight Conformer achieved superior AUC scores in 7 out of 8 datasets, with improvements such as PathMNIST (0.991 vs. 0.990), DermaMNIST (0.961 vs. 0.920), and BloodMNIST (0.999 vs. 0.998). Similarly, we observed considerable improvements in accuracy: 98.82% on PathMNIST compared to 91.1% for ResNet variants, and 99.12% on BloodMNIST compared to 96.6% for Google AutoML Vision. These improvements are especially significant in clinical applications, where increased diagnostic accuracy is critical. The lightweight Conformer consistently outperformed both vanilla CNN-based architectures and advanced AutoML solutions, confirming that our hybrid approach—combining local feature extraction with global attention—is well-suited for medical image analysis tasks.

## 8. Conclusions and Future Scope

Using a hybrid lightweight Conformer-based architecture, this project examined a state-of-the-art deep learning technique for medical image classification. Utilising the advantages of both architectures—convolutional neural networks (CNNs) for capturing fine-grained local patterns and transformers for modelling long-range global dependencies—the suggested model successfully blends CNNs with transformer-based attention mechanisms. The architecture uses ResNet-style bottlenecks and fine-tuned attention layers to further enhance performance, allowing for robust feature extraction from intricate medical images and deeper information propagation. The proposed lightweight Conformer model has consistently surpassed current state-of-the-art methods, including several ResNet variations, AutoML methods, and even transformer models, based on an experimental assessment conducted over a broad collection of benchmark medical imaging datasets such as PathMNIST, DermaMNIST, BloodMNIST, OrganAMNIST, etc. The experimental results demonstrate that the proposed lightweight Conformer architecture achieves competitive performance across multiple medical image classification datasets. Compared with conventional CNNs and transformer-based models, the proposed framework provides a favourable balance between accuracy and computational efficiency.

Additionally, integrating Explainable AI (XAI) techniques such as Grad-CAM, Integrated Gradients, or attention-based visualisations into the lightweight Conformer pipeline can greatly enhance the model’s interpretability. In critical medical settings, providing clinicians with visual justifications for predictions fosters greater trust in AI systems, encourages collaborative decision-making, and aids in validating the model’s outcomes. This will not only improve clinical transparency and accountability but also accelerate the real-world adoption of AI-powered diagnostic systems.

## Figures and Tables

**Table 1:** Summary of datasets used in this study from the MNIST.

Dataset	No. of Images	Classes	Modality
PathMNIST	107,180	9	Histopathology Images
ChestMNIST	112,120	14	Chest X-ray Images
DermaMNIST	10,015	7	Dermatoscopic Images
OCTMNIST	109,309	4	Optical Coherence Tomography (OCT)
PneumoniaMNIST	5856	2	Chest X-ray Images
BloodMNIST	17,092	8	Blood Cell Microscopy Images
RetinaMNIST	1600	5	Fundus Retinal Images
OrganAMNIST	73,600	11	Axial MRI Slices
OrganCMNIST	73,600	11	Coronal MRI Slices
OrganSMNIST	73,600	11	Sagittal MRI Slices

**Table 2:** Architectural comparison between the original Conformer and the proposed lightweight Conformer.

Feature	Original Conformer	Proposed Lightweight Conformer
Network Depth	12+ Transformer Blocks	6–8 Transformer Blocks
Embedding Dimension	384–768	192
Attention Heads	12–16	3–4
MLP Ratio	4.0	2.0
ConvBlock Design	Complex bottleneck with expansion factor of 4	Basic residual design without bottleneck
Convolution Type	Standard Convolution	Depthwise Separable Convolution
Parameters	27.4 M	12.4 M
FLOPs	8.5 G	3.2 G
Inference Time	25.1 ms	10.4 ms

**Table 3:** Architecture configuration of the proposed lightweight Conformer.

Stage	Output Size	Layers	Embedding Dim.	Attention Heads
Input	224×224×3	–	–	–
Stem Conv	112×112×64	1	–	–
Stage 1	56×56	2 Conv Blocks	64	–
Stage 2	28×28	2 Conv Blocks	128	–
Stage 3	14×14	4 Conformer Blocks	192	4
Global Pooling	1×1	–	192	–
Classification Head	Number of Classes	Fully Connected	–	–

**Table 4:** Hyperparameter configuration used for model training.

Hyperparameter	Value
Input Image Size	224×224
Batch Size	32
Number of Epochs	50
Optimizer	AdamW
Learning Rate	$1\times 10^{-4}$
Weight Decay	$1\times 10^{-5}$
Learning Rate Scheduler	Cosine Annealing
Dropout Rate	0.2
Loss Function	Cross Entropy Loss
Activation Function	GELU
Attention Heads	4
Embedding Dimension	192

**Table 5:** Image preprocessing and augmentation procedures.

Operation	Description
Image Resizing	224×224 pixels
Normalization	Pixel values scaled to [0, 1]
Mean-Std Normalization	ImageNet mean and standard deviation
Noise Removal	Basic image denoising and cropping
Horizontal Flip	Probability = 0.5
Vertical Flip	Probability = 0.5
Rotation	$\pm 15^{{}^{\circ}}$
Random Zoom	Range = 0.8–1.2
Random Crop	Applied during training
Color Jitter	Brightness and contrast adjustment

**Table 6:** Dataset characteristics and experimental setup.

Dataset	Images	Classes	Train	Validation	Test
PathMNIST	107,180	9	89,996	10,004	7180
ChestMNIST	112,120	14	78,468	11,219	22,433
DermaMNIST	10,015	7	7007	1003	2005
OCTMNIST	109,309	4	97,477	10,832	1000
PneumoniaMNIST	5856	2	4708	524	624
BloodMNIST	17,092	8	11,959	1712	3421
RetinaMNIST	1600	5	1080	120	400
OrganAMNIST	73,600	11	34,561	6491	32,548
OrganCMNIST	73,600	11	13,000	2392	58,208
OrganSMNIST	73,600	11	13,940	2452	57,208

**Table 7:** Five-fold cross-validation results on the ROP dataset.

Fold	ACC (%)	Precision (%)	Recall (%)	F1-Score (%)	AUC
1	92.8	91.9	93.4	92.6	0.975
2	93.4	92.8	94.0	93.3	0.979
3	94.1	93.6	94.8	94.2	0.982
4	93.2	92.5	93.8	93.1	0.977
5	94.5	93.9	95.1	94.4	0.984
Mean ± SD	93.6±0.68	92.9±0.77	94.2±0.66	93.5±0.67	0.979±0.004

**Table 8:** Five-fold cross-validation results on the BloodMNIST dataset.

Fold	ACC (%)	Precision (%)	Recall (%)	F1-Score (%)	AUC
1	98.9	98.8	98.9	98.8	0.998
2	99.1	99.0	99.1	99.0	0.999
3	99.3	99.2	99.3	99.2	0.999
4	98.8	98.7	98.8	98.7	0.998
5	99.0	98.9	99.0	98.9	0.999
Mean ± SD	99.02±0.19	98.92±0.19	99.02±0.19	98.92±0.19	0.9986±0.0005

**Table 9:** Five-fold cross-validation results on the RetinaMNIST dataset.

Fold	ACC (%)	Precision (%)	Recall (%)	F1-Score (%)	AUC
1	56.7	55.8	56.1	55.9	0.748
2	58.2	57.5	57.9	57.7	0.754
3	59.1	58.4	58.8	58.6	0.760
4	57.8	57.0	57.4	57.2	0.752
5	58.5	57.8	58.2	58.0	0.751
Mean ± SD	58.1±0.91	57.3±0.95	57.7±0.95	57.5±0.94	0.753±0.004

**Table 10:** Comparison of accuracy across datasets with our proposed Conformer model.

Dataset Name	Accuracy Score
BloodMNIST	0.9912
PathMNIST	0.9882
OrganCMNIST	0.9275
OrganAMNIST	0.8571
OCTMNIST	0.8205
PneumoniaMNIST	0.8125
OrganSMNIST	0.8028
Chest-Xray-Pneumonia	0.7997
DermaMNIST	0.7865
BreastMNIST	0.7308
Skin Cancer MNIST-HAM10000	0.6766
RetinaMNIST	0.58

**Table 11:** Computational complexity comparison.

Model	Parameters (M)	FLOPs (G)	Inference Time (ms)
ResNet50	25.6	4.1	12.5
EfficientNet-B0	5.3	0.39	9.8
Vision Transformer	86.5	17.6	25.1
Swin-Tiny	28.3	4.5	13.2
ConvNeXt-Tiny	28.6	4.3	12.9
**Proposed Conformer**	**12.4**	**3.2**	**10.4**

**Table 12:** Comparison of accuracy across models using ROP dataset.

Model	Accuracy (%)
MedVit	85.29
Vision Transformer	84.09
Proposed Lightweight Conformer Model	93.61

**Table 13:** Performance comparison across PathMNIST and DermaMNIST datasets.

Methods	PathMNIST		DermaMNIST	
	AUC	ACC	AUC	ACC
ResNet-18 (28)	0.983	0.907	0.917	0.735
ResNet-18 (224)	0.989	0.909	0.920	0.754
ResNet-50 (28)	0.990	0.911	0.913	0.735
ResNet-50 (224)	0.989	0.892	0.912	0.731
auto-sklearn	0.934	0.716	0.902	0.719
AutoKeras	0.959	0.834	0.915	0.749
Google AutoML Vision	0.944	0.728	0.914	0.768
**Lightweight Conformer Model**	**0.991**	**0.9882**	**0.9609**	**0.768**

**Table 14:** Performance comparison across OCTMNIST and BloodMNIST datasets.

Methods	OCTMNIST		BloodMNIST	
	AUC	ACC	AUC	ACC
ResNet-18 (28)	0.943	0.743	0.998	0.958
ResNet-18 (224)	0.958	0.763	0.998	0.963
ResNet-50 (28)	0.952	0.762	0.997	0.956
ResNet-50 (224)	0.958	0.776	0.997	0.950
auto-sklearn	0.887	0.601	0.984	0.878
AutoKeras	0.955	0.763	0.998	0.961
Google AutoML Vision	0.963	0.771	0.998	0.966
**Lightweight Conformer Model**	**0.9751**	**0.8205**	**0.999**	**0.9912**

**Table 15:** Performance comparison across OrganCMNIST and PneumoniaMNIST datasets.

Methods	OrganCMNIST		PneumoniaMNIST	
	AUC	ACC	AUC	ACC
ResNet-18 (28)	0.992	0.900	0.944	0.854
ResNet-18 (224)	0.994	0.920	0.956	0.864
ResNet-50 (28)	0.992	0.905	0.948	0.854
ResNet-50 (224)	0.993	0.911	0.962	0.884
auto-sklearn	0.976	0.829	0.942	0.855
AutoKeras	0.990	0.879	0.947	0.878
Google AutoML Vision	0.988	0.877	0.991	0.946
**Lightweight Conformer Model**	**0.9936**	**0.920**	**0.9643**	**0.8125**

**Table 16:** Performance comparison across RetinaMNIST and OrganSMNIST datasets.

Methods	RetinaMNIST		OrganSMNIST	
	AUC	ACC	AUC	ACC
ResNet-18 (28)	0.717	0.524	0.972	0.782
ResNet-18 (224)	0.710	0.493	0.974	0.778
ResNet-50 (28)	0.726	0.528	0.972	0.770
ResNet-50 (224)	0.716	0.511	0.975	0.785
auto-sklearn	0.690	0.515	0.945	0.672
AutoKeras	0.719	0.503	0.974	**0.813**
Google AutoML Vision	0.750	0.531	0.964	0.749
**Lightweight Conformer Model**	**0.753**	**0.58**	**0.9780**	0.7902

**Table 17:** Performance comparison with state-of-the-art models.

Model	Accuracy	Precision	Recall	F1 Score	AUC
ResNet50	0.911	0.904	0.899	0.901	0.942
EfficientNet-B0	0.923	0.915	0.912	0.913	0.954
MobileViT	0.927	0.920	0.918	0.919	0.957
DeiT-Tiny	0.931	0.925	0.922	0.923	0.960
Swin-Tiny	0.936	0.931	0.928	0.929	0.965
ConvNeXt-Tiny	0.939	0.934	0.932	0.933	0.968
**Proposed Lightweight Conformer**	**0.946**	**0.941**	**0.939**	**0.940**	**0.972**

**Table 18:** Comparison with recent medical imaging architectures on the ROP dataset.

Model	Accuracy (%)	Parameters (M)	FLOPs (G)
ResNet50	88.7	25.6	4.1
DenseNet121	89.8	8.0	2.9
EfficientNetV2-S	91.4	21.5	8.4
Vision Transformer	90.9	86.5	17.6
Swin-Tiny	92.1	28.3	4.5
TransUNet	91.8	105.3	21.4
MedicalNet	90.7	33.2	6.7
MedViT	92.9	25.1	5.4
**Proposed Lightweight Conformer**	**93.6**	**12.4**	**3.2**

**Table 19:** Ablation study of the proposed architecture.

Model Variant	Accuracy
CNN only	0.882
Transformer only	0.891
CNN + Transformer (baseline Conformer)	0.912
+ Depthwise separable convolution	0.928
+ Reduce Attention	93.1
+ Pretrained weights	0.937
+ Data augmentation	**0.946**

**Table 20:** Evaluation metrics on the ROP dataset.

Metric	Value
Accuracy	93.61%
Precision	92.45%
Recall (Sensitivity)	94.12%
Specificity	91.87%
F1 Score	93.28%
AUC	0.98

## Data Availability

The data presented in this study are openly available in Kaggle at https://www.kaggle.com/datasets/skydevour/medmnist, accessed on 23 June 2026.
